# A Multidisciplinary Integrated Approach for the Identification and Characterization of the AMP Profile in *Hermetia illucens* Hemolymph

**DOI:** 10.3390/insects17050486

**Published:** 2026-05-09

**Authors:** Federica De Stefano, Vittoria Monaco, Fabiana Giglio, Carmen Scieuzo, Roberta Rinaldi, Rosanna Salvia, Gianluca Quaranta, Sofia Amaro, Alessandra Fusco, Ignazio Marcello Mancini, Maria Monti, Giovanna Donnarumma, Patrizia Falabella

**Affiliations:** 1 Department of Basic and Applied Sciences, University of Basilicata, 85100 Potenza, Italy; 2 Department of Chemical Sciences, University of Naples Federico II, 80126 Naples, Italy; 3 CEINGE Advanced Biotechnologies “Franco Salvatore”, 80131 Naples, Italy; 4 Spinoff XFlies S.r.l., University of Basilicata, 85100 Potenza, Italy; 5 Department of Laboratory and Haematological Sciences, Fondazione Policlinico Universitario A. Gemelli Istituto di Ricovero e Cura a Carattere Scientifico (IRCCS), 00165 Rome, Italy; 6 Department of Experimental Medicine, University of Campania “Luigi Vanvitelli”, 80138 Naples, Italy; 7 Department of Life Sciences, Health and Health Professions, Link Campus University, 00165 Rome, Italy; 8 Department of Engineering, University of Basilicata, 85100 Potenza, Italy

**Keywords:** antimicrobial peptides, antimicrobial resistance, *Hermetia illucens*, in vitro antibacterial assay, in silico prediction, proteomics

## Abstract

The growing threat of antimicrobial resistance necessitates alternative strategies to conventional antibiotics. Insects represent a promising source of antimicrobial peptides (AMPs) due to their potent innate immune responses. In this study, we investigated the hemolymph peptide extracts from *Hermetia illucens* larvae as a bioresource of infection-induced AMPs. Larvae were challenged with Gram-negative (*Escherichia coli*) and Gram-positive (*Micrococcus flavus*) bacteria, and hemolymph-derived peptides were extracted and fractionated by RP-HPLC. Peptide fractions were screened in vitro by agar diffusion assay against a panel of pathogenic strains. While extracts from uninfected larvae were essentially inactive, fractions 9–13 from infected larvae showed broad-spectrum antibacterial activity, including against multidrug-resistant clinical isolates such as carbapenem-resistant *Klebsiella pneumoniae*, suggesting an inducible immune response. Fractions were further analyzed by SDS-PAGE and LC-MS/MS to identify candidate AMPs, while shotgun proteomics on unfractionated extracts enabled quantitative comparison among conditions. Proteomics raw data are available via ProteomeXchange Consortium, with the dataset identifier PXD071144. In silico prediction using ProP 1.0, APD3, and CAMP_R4_ supported the antimicrobial potential of candidate peptides. Overall, our integrative approach demonstrates that bacterial infection induces a diverse and bioactive immunopeptidome in *H. illucens*, supporting its potential as a sustainable platform for the discovery of novel antimicrobial agents.

## Simple Summary

Antibiotic resistance is making many bacterial infections harder to treat, so there is an urgent need to identify new antimicrobial substances. Insects represent a promising source of these molecules as they rely on strong natural defenses to survive in environments rich in microorganisms. In this study, we analyzed the hemolymph of Black Soldier Fly larvae (*Hermetia illucens*) to identify the natural molecules produced following a bacterial infection. We compared larvae exposed to various bacteria with untreated larvae and observed that infection induces the production of a wider range of protective molecules. Some of these molecules demonstrated activity against various pathogenic bacteria, including strains that are difficult to treat with currently available antibiotics. By combining antibacterial tests, proteomic analysis, and computational tools, we have identified several candidate molecules that may contribute to the insect’s immune defense. These findings expand our understanding of how *H. illucens* larvae combat infections and highlight this species as a sustainable source of new antimicrobial compounds. In the future, these molecules could facilitate the development of alternative treatments against drug-resistant bacteria, with potential benefits for human health and biotechnology.

## 1. Introduction

In recent years, antimicrobial resistance (AMR) has become a major public health threat and has prompted research into alternative therapeutic agents capable of overcoming the resistance mechanisms of conventional antibiotics [1,2]. Among these, antimicrobial peptides (AMPs) have attracted increasing attention due to their broad-spectrum activity and unique mechanisms of action, including membranolytic and non-membranolytic, which may help prevent antimicrobial resistance [3,4]. Insects, which rely solely on innate immunity, produce a wide range of AMPs, many of which have shown promising biological activity against bacterial, fungal, and viral pathogens [5,6,7,8].

*Hermetia illucens* L. (Diptera: Stratiomyidae), also known as Black Soldier Fly (BSF), has emerged as a particularly rich source of AMPs, thanks to its unique ecological niche, which exposes it to a continuous microbial pressure throughout its life cycle. In particular, *H. illucens* possesses both constitutive and inducible immune mechanisms and is capable of expressing over 50 genes encoding AMPs, some of which are modulated following bacterial infection of larvae [9,10,11]. These peptides belong to different families, such as defensins, cecropins, and attacins, and have demonstrated remarkable efficacy against various clinically relevant pathogens, including multidrug-resistant strains, such as carbapenem-resistant *Klebsiella pneumoniae* [12,13,14]. In addition to their antimicrobial activity, peptide extracts from *H. illucens* have also shown immunomodulatory properties and potential anticancer effects, suggesting a versatile and promising functional profile [15,16,17,18].

In recent years, advanced omics technologies, such as transcriptomic and proteomic approaches, have contributed significantly to the discovery and characterization of insect-derived AMPs by enabling, through RNA sequencing and mass spectrometry, the identification of novel peptide sequences with potential antimicrobial, antiviral, and anticancer properties [8,19].

This study aims to investigate the peptide profile of extracts from *H. illucens* following immune stimulation with Gram-negative and Gram-positive bacteria. Peptide fractions obtained from both infected and uninfected larvae were subjected to high-performance liquid chromatography (HPLC) combined with proteomic analysis to achieve a detailed characterization of their peptide composition. Our approach integrates immune stimulation of larvae with peptide identification by mass spectrometry and the in vitro evaluation of antimicrobial activity against a panel of pathogenic strains, including antibiotic-resistant strains. *E. coli* and *M. flavus* were selected as non-pathogenic, well-characterized reference strains of the Gram-negative and Gram-positive lineages, widely used as immune elicitors in insect models and already employed in our previous work on *H. illucens* hemolymph [11], ensuring consistency and direct comparability with our earlier dataset. In parallel, the peptide sequences identified by proteomic analysis were subjected to a thorough in silico prediction of their structural and functional characteristics, such as physicochemical properties and antimicrobial potential. The integration of experimental and computational methodologies not only provided deeper insights into the structural diversity of AMPs derived from *H. illucens*, but also facilitated the foundation for a rational selection of the most promising candidates for future functional validation studies. This multidisciplinary strategy supports the potential of *H. illucens* as a sustainable source of next-generation antimicrobial peptides.

## 2. Materials and Methods

### 2.1. Hermetia illucens Rearing

*H. illucens* larvae were supplied by Xflies s.r.l (Potenza, Italy). Upon egg hatching, the larvae were reared on a standard Gainesville diet, consisting of 30% alfalfa, 50% wheat bran, and 20% cornmeal [20], with a moisture content of 70%. The rearing process was conducted under controlled environmental conditions: a temperature of 27 ± 1 °C, relative humidity of 70 ± 5%, and a photoperiod of 12 h light and 12 h dark (12L:12D) [21].

### 2.2. H. illucens Larval Infection and Collection of Hemolymph

Larval immune induction was performed following the capillary-immersion procedure established in our previous study on *H. illucens* peptide extracts [11]. *Escherichia coli* (LMG 2092) and *Micrococcus flavus* (DSM 19079), were selected and used as non-pathogenic reference strains representative of the Gram-negative and Gram-positive lineages, respectively, and already validated as AMP elicitors in *H. illucens* larvae [11]. Bacteria were cultured in 10 mL of Luria–Bertani (LB) broth (1% tryptone, 0.5% yeast extract, 0.5% NaCl) at 37 °C for 24 h with shaking at 270 rpm in an Innova 42 incubator shaker (Eppendorf, Hamburg, Germany). 1 mL of each bacterial culture was inoculated into fresh LB broth, incubated at 37 °C, and utilized for the experiment once the optical density (OD) at 600 nm reached ~1, corresponding to late-exponential/early-stationary phase (~10^8^–10^9^ CFU/mL), to ensure a reproducible metabolic state of the inoculum. From these cultures, the bacterial suspensions used for larval immune challenge were then adjusted to 4.0 McFarland (~12 × 10^8^ CFU/mL). Fifth-instar *H. illucens* larvae (n = 100 per condition) were surface-rinsed with sterile water and pricked with a sterile capillary pre-loaded with the bacterial suspension to induce the production of antimicrobial peptides (AMPs) [22,23]. A parallel cohort of uninfected larvae served as the control group. Larvae were kept at 27 °C for 24 h, after which hemolymph was collected from pierced abdomens into ice-cold tubes containing 0.015 g L-ascorbic acid (Merck Millipore) to prevent melanization. Plasma was separated by centrifugation at 10,000 rpm for 5 min at 4 °C using an Eppendorf 5427 R centrifuge (Eppendorf, Hamburg, Germany).

### 2.3. Isolation of Peptide Extract Using Organic Solvents

Low-molecular-weight peptides were isolated from hemolymph plasma by organic-solvent precipitation, as described in Scieuzo et al., 2023 [11]. Plasma was mixed 1:9 (*v*/*v*) with a methanol/acetic acid/water (90:1:9, *v*/*v*/*v*) solution and centrifuged at 13,000 rpm for 45 min at 4 °C using an Eppendorf 5427 R centrifuge (Eppendorf, Hamburg, Germany). The supernatant, enriched in <30 kDa species, was vacuum-dried, resuspended in sterile water (half the initial plasma volume, final concentration 2X), and delipidated by adding an equal volume of hexane, followed by vortexing and centrifugation at 13,000 rpm for 20 min at 4 °C in the same instrument.

### 2.4. Bradford Assay for Protein Quantification

Peptide extract concentrations were measured using the Bio-Rad Protein Assay, Dye Reagent Concentrate (Bio-Rad, Hercules, CA, USA), following the Bradford method [24]. A standard calibration curve using known amounts of Bovine Serum Albumin (BSA) (Merck KGaA, Darmstadt, Germany) was established to determine protein concentration. The sample absorbance was measured at 595 nm using a spectrophotometer (Thermo Scientific, Waltham, MA, USA).

### 2.5. Protein Fractionation by Reverse Phase HPLC

Peptide extracts from both infected and uninfected *H. illucens* larvae were fractionated via reverse-phase HPLC (Agilent Technologies 1100, Santa Clara, CA, USA) using an analytical column (Jupiter 5 µm C18 300 Å, 250 mm × 4.60 mm). The elution was performed using a linear gradient, increasing from 5% to 95% of solvent B (0.07% TFA in 95% acetonitrile) in A (0.1% TFA in HPLC grade water) over 50 min, with a flow rate of 1 mL/min. All fractions were dried using a vacuum concentrator (Thermo Scientific, Waltham, MA, USA) and then stored at −20 °C until further use.

### 2.6. Bacterial Strains and Culture Conditions

The bacterial panel used for antimicrobial testing included reference strains and clinical isolates. *E. coli* (LMG 2092) and *M. flavus* (DSM 19079) were cultured on Luria–Bertani broth/agar (Oxoid Ltd., Basingstoke, UK); *Pseudomonas aeruginosa* (ATCC^®^ 9027^™^) in Brain Heart Infusion broth/agar (Oxoid Ltd.); *Staphylococcus aureus* (ATCC^®^ 6538^™^)*, Salmonella enterica* subsp. enterica serovar Typhimurium (ATCC^®^ 14028GFP^™^)*,* Enteroinvasive *Escherichia coli* (EIEC) (ATCC^®^ 43893^™^)*, Enterococcus faecalis* (ATCC^®^ 29212^™^)*,* and a carbapenem-resistant *Klebsiella pneumoniae* (CRKP) in Tryptic Soy broth/agar (Oxoid Ltd.). The CRKP strain was clinically isolated at the Unit of Microbiology and Virology of the University of Campania “Luigi Vanvitelli”.

### 2.7. Evaluation of the Antibacterial Activity of HPLC Fractions via Agar Diffusion Assay

The antibacterial activity of HPLC fractions was evaluated in vitro using the agar diffusion assay. A single colony of each bacterial strain was inoculated into the appropriate culture medium and incubated overnight at 37 °C with continuous shaking at 270 rpm in an Innova 42 incubator shaker (Eppendorf, Hamburg, Germany). Subsequently, 100 μL of each bacterial suspension, exhibiting an optical density of 0.3 at 600 nm (OD600, approximately 10^8^ CFU/mL), was spread on a solid medium using a sterile cotton swab. After the bacterial suspension was absorbed, 5 μL of each HPLC fraction, from both infected and uninfected larvae, was spotted in duplicate on the plates. Sterile water was used as a negative control. Plates were incubated overnight at 37 °C [25]. Scoring of the agar diffusion panels was performed qualitatively by visual inspection, following criteria consistent with established standards for agar diffusion testing [26] and with published reviews on antimicrobial susceptibility testing of natural products and peptide extracts [25,27]. Each spot was scored as: “inhibition” when a clear area of absent bacterial growth, with a well-defined outer margin and extending beyond the edge of the deposited droplet, was visible over the surrounding bacterial lawn; and “no inhibition” when no clearance was observed, or when only a drop imprint (i.e., a wetting or precipitation mark circumscribed to the area of the deposited droplet and lacking a defined outer margin of growth suppression) was visible. Faint or hazy growth at the edge of the clearance area, if any, was disregarded, in line with CLSI M02 guidance for visual reading of diffusion plates. As explicitly stated below, the assay was used as a qualitative screening tool to identify active fractions, and inhibition zone diameters were not used for quantitative potency comparisons.

### 2.8. SDS-PAGE, in Situ Hydrolysis and Protein Identification Procedure

HPLC fractions obtained from peptide extracts of *H. illucens* larvae infected with *E. coli*, *M. flavus*, or from uninfected larvae were separated using sodium dodecyl sulfate-polyacrylamide gel electrophoresis (SDS-PAGE). Specifically, for each fraction, 20 µL of a 1X loading buffer was added, which consisted of 2% SDS (Bio-Rad, Hercules, CA, USA), 50 mM Tris-HCl pH 6.8 (Merck KGaA, Darmstadt, Germany), 10% glycerol (Merck KGaA, Darmstadt, Germany), and bromophenol blue (Bio-Rad, Hercules, CA, USA). Samples were resolved on a 20% SDS-PAGE gel. A pre-stained molecular weight marker was loaded in parallel to estimate the molecular size of resolved bands. The markers used were Precision Plus Protein™ Standards All Blue (Bio-Rad, Cat. No. 161-0373, Hercules, CA, USA) and ProSieve^®^ QuadColor™ Protein Markers (Lonza Rockland, Inc., Cat. No. 00193837, Rockland, ME, USA). After electrophoresis, the gel was stained with GelCode™ Blue Safe Protein Stain (Thermo Fisher Scientific, Waltham, MA, USA) and then destained in Milli-Q water [28]. Fifteen bands from HPLC fractions of peptide extracts from uninfected larvae, twenty-one bands from fractions obtained from larvae infected with *E. coli*, and twenty-three bands from fractions obtained from larvae infected with *M. flavus* were excised and subjected to in situ hydrolysis with trypsin, as previously described [29]. Peptide mixtures were extracted using a solution of 0.2% formic acid (HCOOH) in acetonitrile (ACN) and subsequently dried with a vacuum concentrator (Thermo Scientific, Waltham, MA, USA).

The peptide mixtures were then analyzed by LC-MS/MS using an Orbitrap Exploris 240 mass spectrometer equipped with a Nanospray Flex ion source and coupled with a Vanquish Neo nanoUPLC system. Samples from shotgun and in situ hydrolyses were fractionated on a C18 capillary reverse phase column (150 mm, 75 μm, 2 μm 100 Å) at a flow rate of 250 nL/min. A linear gradient of eluent B (0.2% formic acid in 95% acetonitrile) in A (0.2% formic acid and 2% acetonitrile in LC-MS grade water) was used from 2% to 90% in 30 min and 77 min for the SDS-PAGE and shotgun analyses, respectively. The fragmentation was performed on the top 30 ions in a Data-Dependent Acquisition (DDA) mode with a dynamic exclusion time of 25 s or 40 s for the SDS-PAGE and shotgun analysis respectively. Protein identification was performed using Proteome Discoverer 2.5 software with a specific *H. illucens* protein database. Proteins with at least 10% sequence coverage were considered.

### 2.9. Shotgun Differential Proteomics Analysis

20 µg of peptide extract from three biological replicates from *H. illucens* uninfected larvae, infected with *E. coli* or with *M. flavus* were hydrolyzed with a shotgun approach by employing the S-Trap cartridges (Protifi), as previously described [30]. Each peptide mixture was analyzed in duplicate by LC-MS/MS and proteins identified by Proteome Discoverer 2.5. The statistical analysis of the protein output (normalized abundances) from the differential proteomics approach was performed by using the Perseus 1.6.15.0 software. Differences were detected by applying a one-way ANOVA statistical test followed by post hoc Tukey’s HSD [31,32]. Briefly, only proteins that did not present zero values in any replicate at least in one condition were considered. Furthermore, in the Perseus imputation procedure, each zero was corrected with the minimum value of the log2 determined for the normalized intensities (i.e., 2.6). Proteins having at least 10% sequence coverage and statistically validated data (FDR < 0.05) were considered.

### 2.10. In Silico Analysis for Evaluation of Physicochemical Properties and for Antimicrobial Activity Prediction

The sequences identified by mass spectrometry were analyzed using the ProP 1.0 (https://services.healthtech.dtu.dk/services/ProP-1.0/) (accessed on 7 March 2026) server to identify the signal peptide cleavage site and the pro-peptide region [33]. The mature and active regions of the peptides were then analyzed in silico to evaluate physicochemical properties such as peptide length, molecular weight, total hydrophobic ratio, total net charge, isoelectric point, and Boman index. These parameters were determined using the Antimicrobial Peptide Database Calculator and Predictor (https://aps.unmc.edu/prediction) (APD6) (accessed on 7 March 2026) [34,35,36] and the Compute pI/Mw—Expasy tool (https://web.expasy.org/compute_pi/) (accessed on 7 March 2026) [37,38,39]. The mature and active regions of the peptides were subsequently analyzed in silico using three machine learning algorithms available in the CAMP_R4_ (https://camp.bicnirrh.res.in/) database (accessed on 7 March 2026) [40]: Random Forest (RF), Support Vector Machine (SVM), and Artificial Neural Network (ANN), to predict their antimicrobial activity. When all sequences were analyzed with the algorithms, those with a score greater than or equal to 0.5 were automatically considered potential antimicrobials by the software. It should be noted that the threshold is set intrinsically by the software and cannot be changed by the user. This applies to all algorithms (RF, SVM, and ANN), which report the result in numerical form (score). Each of these, alongside the numerical score, returns results in categorical form, namely AMP (antimicrobial peptide) or NAMP (non-antimicrobial peptide).

### 2.11. BLAST Search and Multiple Sequence Alignment

Mature peptide sequences identified by LC-MS/MS were used as queries for systematic BLASTp searches against the NCBI non-redundant protein database (https://blast.ncbi.nlm.nih.gov/) (accessed on 19 April 2026) and the UniProtKB database (https://www.uniprot.org/) (accessed on 19 April 2026) using the latest online versions/releases available at the time of analysis and default parameters. Database annotations were updated as of 19 April 2026. For each peptide, the best hit (accession number, description, organism, query cover, percent identity, and e-value), together with the corresponding UniProt annotation and matched protein region whenever available, was reported in Supplementary Tables S3 and S4. Based on the best BLAST and UniProt hits, peptides were assigned to AMP families according to the following criteria: peptides showing significant similarity (≥60% identity over the mature region) to annotated AMPs were classified into the corresponding family; peptides without significant database hits or annotated as uncharacterized/hypothetical proteins were grouped as “unclassified”. Multiple sequence alignments were then performed separately for each family using Clustal Omega, available through the EMBL-EBI Job Dispatcher web server (https://www.ebi.ac.uk/jdispatcher/msa/clustalo) (accessed on 19 April 2026) [41] using the latest online version available at the time of analysis and with default parameters. Alignments were visualized using the default Clustal Omega color scheme.

## 3. Results

### 3.1. Evaluation of Sample Concentration

The concentration of the samples obtained following precipitation with organic solvents from hemolymph was evaluated via the Bradford assay.

The values obtained are shown in the following table (Table 1).

### 3.2. Protein Fractionation by Reverse Phase HPLC

**Figure 1:**
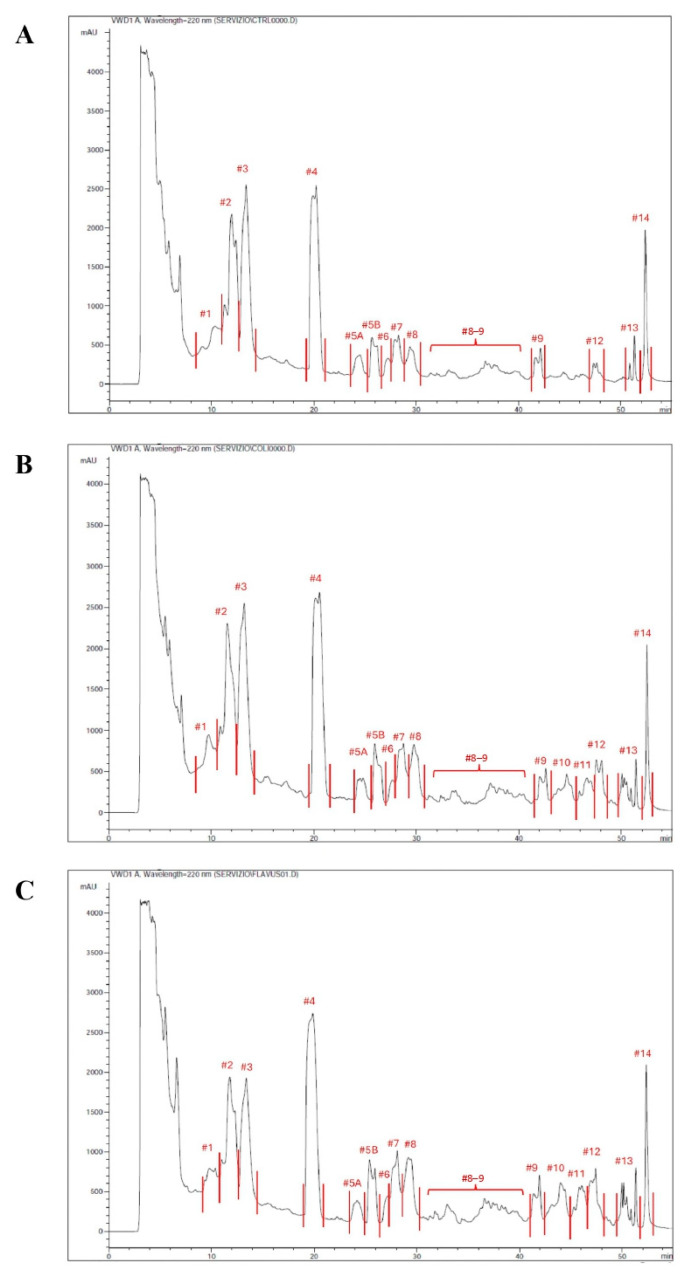
RP-HPLC chromatographic profile of peptide extract and fractions collected by uninfected larvae (**A**), larvae infected with *E. coli* (**B**) and larvae infected with *M. flavus* (**C**). The fraction numbering is consistent across conditions; in the control, fractions 10–11 are missing as peaks were not detected.

The peptide extracts were fractionated via reverse-phase HPLC, resulting in a total of 14 fractions for the peptide extract from uninfected larvae (Figure 1A), 16 fractions for the peptide extract from larvae infected with *E. coli* (Figure 1B), and 16 fractions for the peptide extract from larvae infected with *M. flavus* (Figure 1C).

### 3.3. Evaluation of the Antibacterial Activity of HPLC Fractions via Agar Diffusion Assay

The antimicrobial activity of HPLC fractions obtained from the peptide extract from *H. illucens* larvae was evaluated against eight different bacterial strains: *E. coli*, *M. flavus*, *P. aeruginosa*, *S. aureus*, *E. faecalis*, *S. typhimurium*, EIEC, and CRKP.

Throughout this section, the classification of each HPLC fraction as “inhibition” or “no inhibition” follows the criteria defined in Section 2.7, and representative examples of both categories are provided in Supplementary Figures S1–S8: panels A (HPLC fractions from uninfected larvae) generally show “no inhibition”, while panels B and C from infected larvae provide examples of both “no inhibition” and “inhibition”.

Agar diffusion assay results generally showed no inhibition zones in HPLC fractions from peptide extracts of uninfected larvae. An exception was observed in the results of the agar assays against *E. coli* (Supplementary Figure S1A) and against *M. flavus* (Supplementary Figure S2A), in which certain fractions (fractions between 9 and 14 against *E. coli* and fractions between 8–9 and 14 against *M. flavus*) exhibited activity. For fractions derived from peptide extracts from larvae infected with *E. coli*, inhibition zones were observed in fractions between 9 and 13 against both *E. coli* and *M. flavus*, except for fraction 10 against *M. flavus*. Similarly, in fractions from larvae infected with *M. flavus*, inhibition zones were detected in fractions between 9 and 13 against the *E. coli* and in fractions 11, 12, and 13 against *M. flavus*. Notably, none of the fractions between 1 and 8 exhibited antimicrobial activity against either *E. coli* or *M. flavus* (Supplementary Figures S1B,C and S2B,C).

Agar diffusion assay results against *P. aeruginosa* showed inhibition zones for the HPLC fractions 11, 12 and 13 of peptide extracts from larvae infected with *E. coli* (Supplementary Figure S3B) and stronger inhibition zones for fractions 10, 12 and 13 from peptide extracts from larvae infected with *M. flavus* (Supplementary Figure S3C). Importantly, none of the HPLC fractions from peptide extracts from uninfected larvae showed antimicrobial activity (Supplementary Figure S3A).

Similarly, the assay against *S. aureus* revealed inhibition zones for HPLC fractions 9, 10, 11, 12 and 13 from peptide extracts from larvae infected with *E. coli* (Supplementary Figure S4B), and 9, 11, 12 and 13 from peptide extracts from larvae infected with *M. flavus* (Supplementary Figure S4C), with the strongest activity observed for fractions 12 and 13 in both cases. Only fraction 9 from peptide extracts from uninfected larvae exhibited antimicrobial activity (Supplementary Figure S4A).

In the case of *E. faecalis*, inhibition zones were observed for HPLC fractions 9, 11, 12 and in particular 13 obtained from peptide extracts from larvae infected with both *E. coli* and *M. flavus* (Supplementary Figure S5B,C). As observed in the assay against *P. aeruginosa*, no activity was detected in fractions from peptide extracts from uninfected larvae (Supplementary Figure S5A).

For *S*. Typhimurium, HPLC fractions 12 and 13 from peptide extracts from larvae infected with *E. coli* (Supplementary Figure S6B) and fractions 12, 13 and 14 from peptide extracts from larvae infected with *M. flavus* (Supplementary Figure S6C) produced inhibition zones. However, no activity was detected in the fractions from peptide extracts from uninfected larvae (Supplementary Figure S6A).

The assay against EIEC also showed inhibition zones for HPLC fractions 9, 10, 11, 12, and 13 derived from peptide extracts from larvae infected with *E. coli* (Supplementary Figure S7B), as well as for the fractions 11, 12 and 13 from peptide extracts from larvae infected with *M. flavus* (Supplementary Figure S7C). Also in these cases, the strongest activity was observed in fractions 12 and 13. None of the fractions from the peptide extracts from uninfected larvae exhibited antimicrobial activity in this assay (Supplementary Figure S7A).

Finally, the results of the agar diffusion assay against CRKP revealed inhibition zones for HPLC fractions 9, 10, 12 and 13 obtained from peptide extracts from larvae infected with *E. coli*, as well as for the fractions 11, 12 and 13 from peptide extracts from larvae infected with *M. flavus* (Supplementary Figure S8B,C), with the strongest activity observed in fractions 12 and 13. Consistent with the previous observations, none of the HPLC fractions from peptide extracts from uninfected larvae exhibited antimicrobial activity (Supplementary Figure S8A).

These findings underscore the antimicrobial potential of specific HPLC fractions from peptide extracts from infected larvae, while also revealing limited activity in some fractions from uninfected ones. In line with the qualitative nature of agar diffusion assays, these conclusions are based on the presence or absence of visible inhibition zones, without quantitative measurements of diameters.

### 3.4. SDS-PAGE Separation of the HPLC Fractions

**Figure 2:**
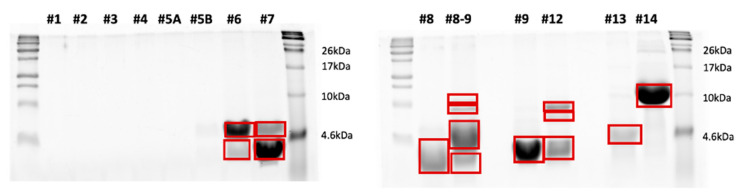
SDS-PAGE separation of the HPLC fraction of peptide extracts by uninfected larvae. The bands highlighted in red were excised from the gel and in situ hydrolyzed.

**Figure 3:**
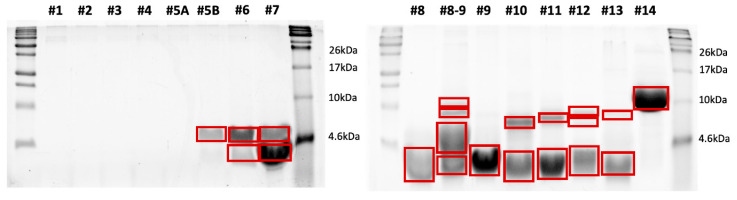
SDS-PAGE separation of the HPLC fraction of peptide extracts by larvae infected with *E. coli*. The bands highlighted in red were excised from the gel and in situ hydrolyzed.

**Figure 4:**
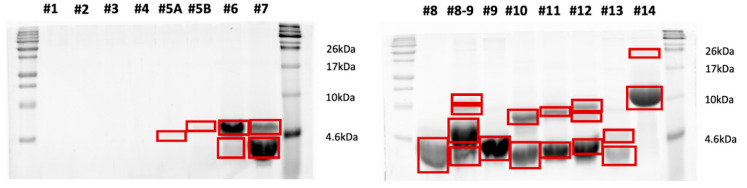
SDS-PAGE separation of the HPLC fraction of peptide extracts by larvae infected with *M. flavus*. The bands highlighted in red were excised from the gel and in situ hydrolyzed.

Following HPLC separation of the peptide extracts, the collected fractions were loaded onto SDS-PAGE gels for analysis. Figure 2, Figure 3 and Figure 4 show the SDS-PAGE separation profiles of HPLC fractions of peptide extracts derived from *H. illucens* larvae under different experimental conditions: uninfected (Figure 2), infected with *E. coli* (Figure 3), and infected with *M. flavus* (Figure 4).

The gel profiles corresponding to fractions of peptide extracts from larvae infected with *E. coli* and *M. flavus* reveal a broader range of distinct bands, compared to the uninfected larvae, indicating the presence of additional peptide species. The quantitative and qualitative differences in band patterns support the hypothesis that infection stimulates the production of immune-related peptides, as evidenced by the increased number and intensity of bands in the HPLC fractions from infected samples.

### 3.5. In Gel Protein Identification

**Figure 5:**
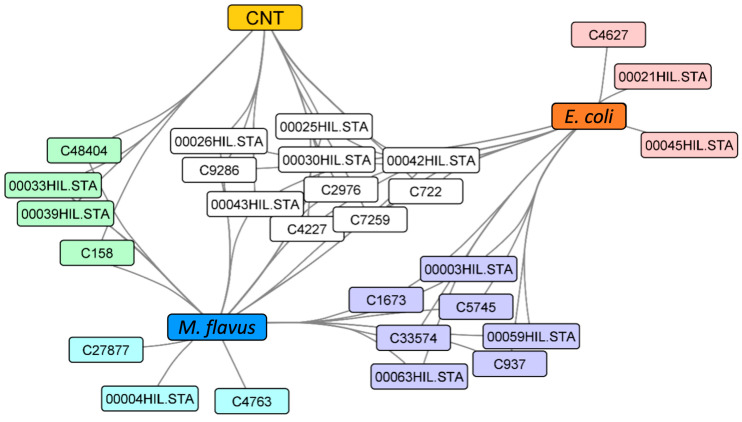
Cytoscape network representation of the proteins identified in *H. illucens* peptide extracts from the three conditions. The color code refers to the differently shared protein sets.

Bands highlighted in the SDS–PAGE gels shown in Figure 2, Figure 3 and Figure 4 were excised and subjected to a classical in situ hydrolysis protocol [42]. The resulting peptide mixtures were directly analyzed by LC-MS/MS. Fragmentation data were used for protein identification using the Proteome Discoverer 2.5 software. The proteins identified under the different experimental conditions are reported in Supplementary Table S1 and graphically summarized in Figure 5, by employing the ClueGO app 2.5.7 on the Cytoscape platform for network representation. As expected, the largest number of identified species were shared among the three samples or between the control and one of the two other samples. However, specific peptides also emerged from each of the two infected conditions.

### 3.6. Differential Proteomics Analysis

Not-fractionated peptide extracts from *H. illucens* harvested under the three experimental conditions (uninfected control, infected with *M. flavus* and infected with *E. coli*) were also analyzed using a shotgun label-free differential proteomics approach, in order to quantitatively evaluate variations in peptide abundance. Briefly, 20 µg of peptide extract from each of the three samples were directly digested with trypsin using an S-Trap cartridge [30], and the peptide mixtures were analyzed by LC-MS/MS.

**Figure 6:**
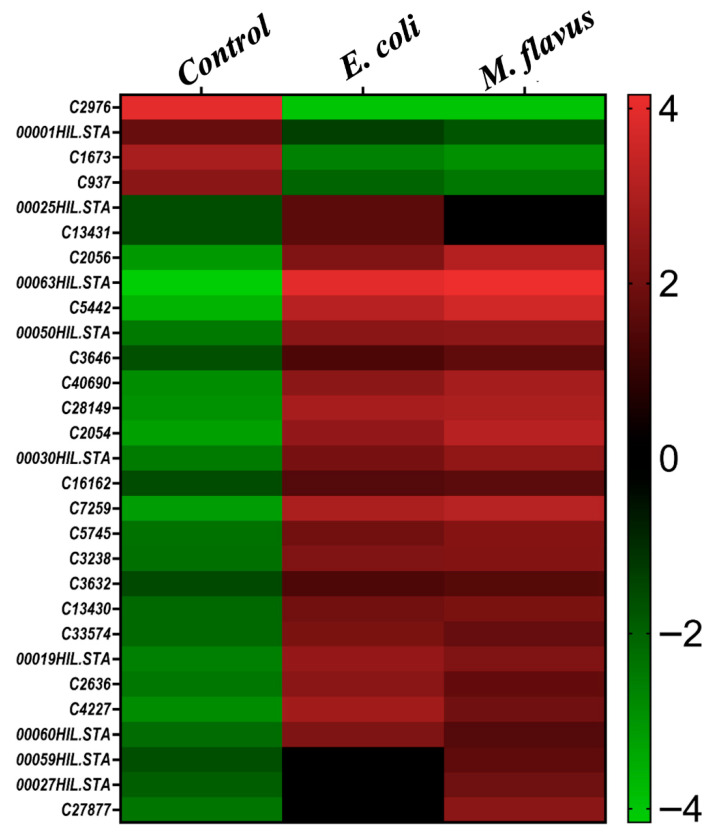
Heatmap representation of the 29 proteins and their relative abundances among the three conditions analyzed by the differential proteomics experiment. Protein relative abundances were reported according to Tukey’s factors (i.e., abundances mean differences between two comparisons) from green (less abundant) to red (highly abundant).

The protein species were identified and quantified with the Proteome Discoverer 2.5 software, and statistical analysis was performed with the Perseus 1.6.15.0 software. Proteins showing significant differences by one-way analysis of variance (ANOVA) were subjected to Tukey’s post hoc test to identify significant pairwise comparisons among the three groups. A total of 29 proteins statistically significant were identified (Supplementary Table S2), and Tukey’s factors determined by the Perseus 1.6.15.0 software were used to generate a heatmap representation reported in Figure 6.

Among these, 20 proteins were up-regulated in peptide extracts from larvae infected with both *E. coli* and *M. flavus*, 2 were up-regulated only in the peptide extract from larvae infected with *E. coli*, 3 were up-regulated only in peptide extracts from larvae infected with *M. flavus*, and 4 were down-regulated in the peptide extracts from both *E. coli* and *M. flavus* infected larvae.

It is important to highlight that the set of peptides identified from the gel bands overlapped significantly with those of the statistically significant proteins (FDR < 0.05) identified in the differential proteomic analysis. In particular the shared proteins were: *C2976, C1673*, *C7259*, *C5745*, *C33574*, *C4227*, *C27877*, *000025HIL.STA*, *000063HIL.STA*, *000030HIL.STA*, *000059HIL.STA*.

### 3.7. In Silico Analysis for Evaluation of Physicochemical Properties and for Antimicrobial Activity Prediction

All identified sequences (27 from mass spectrometry analysis of HPLC fractions and 29 in Differential Proteomics Analysis of not-fractionated peptide extracts) were initially analyzed in silico using ProP 1.0 to identify the signal peptide and pro-peptide. Once the mature sequences were obtained, the physicochemical properties of these peptides were evaluated using the APD3 (Antimicrobial Peptide Database) calculator and predictor (Supplementary Tables S3 and S4). The molecular mass of the peptides ranges from 2689.24 Da for the smallest peptide to 19,262.244 Da for the largest peptide. The amino acid sequences range in length from a minimum of 26 residues to a maximum of 183 residues. The total hydrophobic ratio showed a minimum value of 19% and a maximum value of 58%. The total net charge of the identified peptides ranges from –4.25 to +9.25. The Boman Index identified values ranging from a minimum of 0.44 to a maximum of 3.58 in kcal/mol, while the isoelectric point (pI) ranged from 4.95 to 11.83. In addition, all sequences were analyzed in silico using three machine learning algorithms available in the free online database CAMP_R4_: Random Forest (RF), Support Vector Machine (SVM), and Artificial Neural Network (ANN) in order to predict their antimicrobial activity. The results are reported in Supplementary Tables S3 and S4. Of the sequences identified by mass spectrometry of HPLC fractions, 20 peptides were predicted to be antimicrobial, meeting the criterion of 3 out of 3 parameters ≥0.5, while 18 peptides identified in the differential proteomic analysis were predicted to be antimicrobial in silico. All predicted activities are listed in Supplementary Tables S3 and S4.

### 3.8. Classification of Identified Peptides into AMP Families and Multiple Sequence Alignment

**Figure 7:**
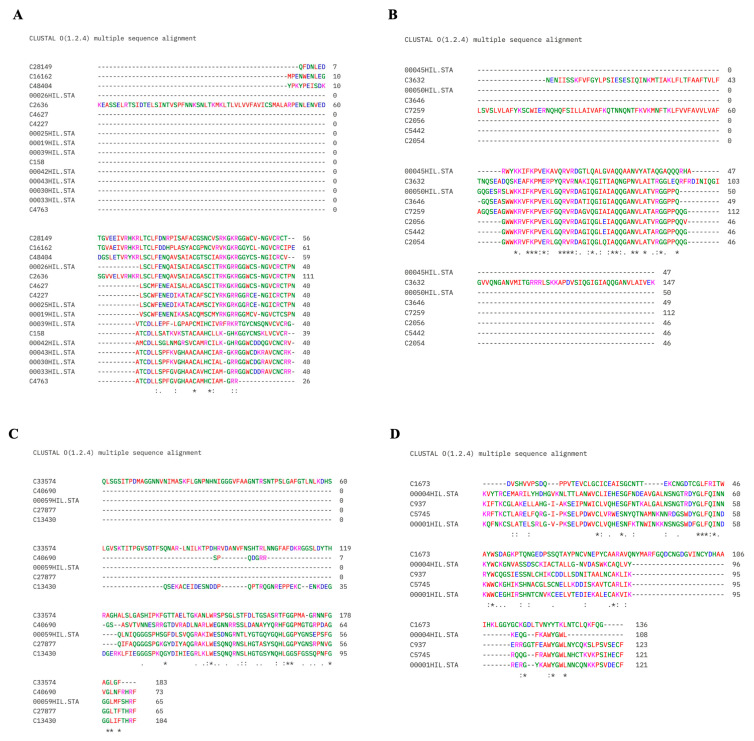
Multiple sequence alignments of *H. illucens* hemolymph peptides grouped into AMP families. Alignments were generated using Clustal Omega from the mature peptide sequences reported in Supplementary Tables S3 and S4, after assignment to AMP families based on BLASTp and UniProt annotations. (**A**): defensin-like peptides; (**B**): cecropin-like peptides; (**C**): attacin-like peptides; (**D**): lysozyme-like proteins. Residues are colored according to the default Clustal X colour scheme. The consensus line below each alignment block uses the standard Clustal Omega notation: an asterisk (*) indicates a position with a single, fully conserved residue (i.e., the same amino acid in all aligned sequences); a colon (:) indicates conservation between residues with strongly similar properties (substitution score >0.5 in the Gonnet PAM250 matrix); a period (.) indicates conservation between residues with weakly similar properties (substitution score ≤0.5 in the Gonnet PAM250 matrix); a blank space indicates no conservation. Contiguous asterisks in the consensus line therefore correspond to consecutive fully conserved positions, each marked by an individual “*” symbol, and do not represent a multi-level scoring system.

To further characterize the peptide repertoire identified in *H. illucens* hemolymph, all mature peptide sequences were re-annotated by systematic BLASTp analysis against the NCBI non-redundant protein database and by UniProtKB searches (see Section 2.11). The complete results (accession, description, identity, e-value, UniProt annotation and assigned family) are reported in the Supplementary Tables S3 and S4. On this basis, the identified peptides could be grouped into four well-defined AMP families (defensin-like, cecropin-like, attacin-like, and lysozyme-like), together with a small set of unclassified peptides corresponding to sequences annotated as uncharacterized or lacking significant database hits. Multiple sequence alignments performed separately for each family using Clustal Omega revealed a high level of intra-family conservation (Figure 7), including the cysteine scaffold and RxKR pro-peptide cleavage site of defensin-like peptides, the WWKR/KPVEK/RVR signature of cecropin-like peptides, the glycine-rich C-terminal motif (GGL[I/M/V]-F[T/S]-HRF) of attacin-like peptides, and the conserved residues typical of invertebrate-type lysozymes.

## 4. Discussion

The results of the present study demonstrate that infection of *H. illucens* larvae with *E. coli* or *M. flavus* stimulates the production of antimicrobial peptides (AMPs) with significant activity against a range of pathogenic bacteria. At the same time, peptide extracts from uninfected larvae also displayed a limited but detectable antimicrobial activity against *E. coli* and *M. flavus*, consistent with the presence of a constitutive baseline of immune effectors already described in the literature [9,11]. Bacterial challenge, however, substantially broadened the spectrum of activity, extending it to additional Gram-negative and Gram-positive pathogens, including the multidrug-resistant, CRKP. This pattern supports a model in which an inducible immune response is superimposed on a constitutive basal defense.

The most pronounced antimicrobial activity was consistently observed in HPLC fractions from 9 to 14 from infected larvae, whereas only limited and strain-dependent activity was detected in the corresponding fractions from uninfected larvae, a pattern further supported by SDS-PAGE analysis showing a broader and more intense band pattern profile following bacterial challenge. Proteomic analysis also revealed a set of differentially abundant proteins across conditions, including shared infection-specific candidates, supporting a pathogen-responsive modulation of the hemolymph peptidome. These findings are consistent with the hypothesis that the BSF deploys a highly inducible and pathogen-responsive antimicrobial system, in which infection triggers a strong activation of effectors that are already present at basal levels under non-infected conditions. The observation that several fractions from infected larvae were active against clinically relevant pathogens and multidrug-resistant bacteria, such as CRKP, underscores their potential as alternative agents in the fight against antimicrobial resistance, one of the most pressing global health challenges [2].

Moreover, the in silico analysis confirmed that many of the identified peptides possess physicochemical properties typical of active AMPs, such as a high net positive charge and moderate hydrophobicity, supporting their ability to interact with and disrupt bacterial membranes. The integration of in silico physicochemical profiling with machine learning-based predictions (CAMP_R4_) further supported the functional annotation of candidate peptides, validating their suitability for membrane interaction and potential bactericidal activity. It is interesting to note that 7 peptides identified in the active fractions were not predicted to be antimicrobial by in silico tools. Similarly, 11 peptides were identified in the total peptide extract with no evidence of predicted antimicrobial activity according to the same in silico predictions. Almost all of these peptides overlap between the two analyses, suggesting that they may represent a context-dependent subclass. This overlap reinforces the hypothesis that these peptides may potentially act synergistically, enhancing the activity of other AMPs present in the peptide extracts.

Notably, some of the identified peptides are already included in established international peptide databases such as the Protein Data Bank and the Antimicrobial Peptide Database (APD3). In particular, peptide C158 (APD ID: AP03308), detected in fractions 8–9 of peptide extracts from unstimulated larvae and larvae stimulated with *M. flavus*, was previously identified and characterized in 2022 by Zhang and colleagues. This peptide, expressed in *E. coli* BL21(DE3) as a fusion protein with thioredoxin, exhibited antimicrobial activity against Gram-positive and Gram-negative bacteria [43].

Another relevant peptide is 00043HIL.STA (APD ID: AP02548), corresponding to DLP4, a defensin-like molecule identified in fraction 9 of peptide extracts from both unstimulated larvae and those stimulated with *E. coli*, and in fraction 8–9 from larvae stimulated with *M. flavus*. Originally isolated from the hemolymph of immunized *H. illucens* larvae [44], DLP4 showed antimicrobial activity specifically against Gram-positive bacteria, including methicillin-resistant *S. aureus* (MRSA), with gene expression particularly induced in the fat body following bacterial challenge. Subsequent studies demonstrated that although native DLP4 displayed moderate antimicrobial activity and some cytotoxicity, a rationally designed derivative, ID13, exhibited significantly stronger activity (MIC 4–8 μg/mL against Gram-positive pathogens) and lower cytotoxicity, as well as membrane-disrupting and DNA-binding properties [45]. Moreover, recombinant DLP4 and its homolog DLP2, expressed in *Pichia pastoris,* showed higher potency, a longer post-antibiotic effect, and better efficacy than vancomycin in vivo models of MRSA infection, without inducing bacterial resistance after serial passage [45]. More recently, Jiang and colleagues established a prokaryotic expression and purification system using an ELP-intein fusion strategy to obtain functional DLP4, further confirming its antimicrobial activity [46]. Together, these findings highlight DLP4 as a promising candidate for therapeutic development against antibiotic-resistant Gram-positive infections.

Another peptide identified in fractions 10 and 12 of the peptide extract from larvae infected with *M. flavus* is diptericin C27877 (APD ID: AP03742), also known as Hill-Dip6, reported in the study by Van Moll et al. (2022) [14], in which it was included in a library of 36 synthetic antimicrobial peptides derived from *H. illucens*. The study represented the first systematic evaluation of the activity of these AMPs against a broad panel of human pathogens (*S. aureus*, *E. coli*, *P. aeruginosa*, *Candida albicans*, and *Aspergillus fumigatus*) and a human cell line (MRC5-SV2) [14]. Although Hill-Dip6 was not among the two peptides selected for in-depth functional analysis, it showed activity against *E. coli* (MIC of 2 μM), and its inclusion in the library highlights the remarkable structural diversity of the black soldier fly AMP repertoire, one of the most extensive recorded in insects [14].

A further noteworthy case concerns peptide 00030HIL.STA (Supplementary Tables S3 and S4), identified in the present study in fractions 11–12 of peptide extracts from both *E. coli*- and *M. flavus*-infected larvae by LC-MS/MS, and consistently recovered also in the differential shotgun proteomic analysis. The mature sequence of 00030HIL.STA (ATCDLLSPFKVGHAACALHCIALGRRGGWCDGRAVCNCRR) shares 100% amino-acid identity over the entire 40-residue mature region with the peptide Hill_BB_C7176, originally predicted in silico from the *H. illucens* transcriptome by Moretta et al. [9] and recently validated experimentally by Derin et al. [47]. In that work, synthetic Hill_BB_C7176 displayed antibacterial activity against *S. aureus* and *E. coli*, a concentration-dependent uptake of propidium iodide consistent with membrane permeabilization, strong binding affinity towards lipopolysaccharide (lipid A), and in vivo efficacy in *Galleria mellonella* infection models comparable to reference antibiotics under the conditions tested. Our proteomic detection of 00030HIL.STA in the hemolymph of bacterially challenged larvae therefore provides the first direct evidence of its endogenous induction during infection, thereby closing the cycle between transcriptome-based in silico prediction [9], bioactive validation of the synthetic peptide [47], and demonstration of its actual biosynthesis upon immune challenge in the live insect. This triangulation strengthens the rationale of integrated experimental–computational pipelines for AMP discovery in *H. illucens* and supports 00030HIL.STA/Hill_BB_C7176 as a promising lead for further translational development.

Beyond the three AMPs for which dedicated characterization studies have been previously published (C158/Hidefensin1, DLP4 and C27877), systematic BLASTp and UniProt annotation of the full set of identified peptides (Figure 7, Supplementary Tables S3 and S4) revealed that the vast majority of sequences belong to well-defined insect AMP families. In particular, 16 peptides were assigned to the defensin family, spanning isoforms of DLP1, DLP4, defensin B-like and defensin-like peptide C-13326, 8 to cecropin-like peptide 1, 5 to attacin-like peptides, and 5 to invertebrate-type lysozymes. In this work, we adopt the more recent annotations available in NCBI and UniProt. The presence of multiple isoforms within each family, together with the conservation of the canonical AMP signatures in the Clustal Omega alignments, supports the evolutionary expansion of defensin, cecropin and attacin gene families in *H. illucens* [9] and suggests that peptide isoforms co-eluting in the same HPLC fractions may contribute cooperatively to the observed antimicrobial activity. A smaller set of unclassified peptides includes cysteine-rich and glycine/chitin-binding-like sequences corresponding to *H. illucens* uncharacterized proteins, which may represent candidates for further functional characterization.

Importantly, the use of a dual proteomic approach, combining shotgun analysis of whole peptide extract with fraction-based LC-MS/MS following SDS-PAGE, allowed the identification of both abundant and low-expression peptides, broadening the spectrum of putative AMPs discovered. This methodological integration proved particularly valuable for uncovering inducible immune effectors that might otherwise be missed in standard proteomic analyses.

The differential response to *E. coli* compared with *M. flavus* infections also suggests a level of pathogen discrimination in the innate immune system of *H. illucens*, potentially mediated by selective signaling pathways or effector gene regulation. This finding opens new avenues for further transcriptomic and functional studies aimed at dissecting the regulatory mechanisms behind AMP induction.

Taken together, our findings indicate that *H. illucens* combines a constitutive basal antimicrobial repertoire with a strongly inducible response upon infection, resulting in a dynamic and adaptable immune system. This dual strategy further supports its potential as a scalable and renewable biotechnological platform for AMP discovery, aligning with global efforts to develop environmentally sustainable and effective solutions against antimicrobial resistance.

## 5. Conclusions

The present study provides comprehensive and multifaceted experimental evidence that infection-induced peptide extracts of hemolymph of *H. illucens* larvae are a rich and dynamic source of antimicrobial peptides (AMPs). Rather than focusing solely on antimicrobial activity, this study emphasizes the combined power of biochemical fractionation, high-resolution proteomics, and in silico prediction tools to identify and prioritize AMP candidates with promising physicochemical characteristics and computational antimicrobial profiles.

While antimicrobial activity against specific pathogens was addressed in previous sections, the present work adds value by demonstrating how a dual proteomic approach—SDS-PAGE-coupled LC-MS/MS and label-free shotgun proteomics—allows a more complete and unbiased mapping of the immunopeptidome. Such integration of empirical and computational workflows enhances the robustness of AMP discovery and paves the way for more targeted validation pipelines.

Notably, different peptides identified in this study overlap with known AMPs reported in international databases, thereby reinforcing the validity and translational relevance of the methodological framework approach adopted.

Beyond the scope of innate immunity, our findings support the broader applicability of insect-derived biomolecules in biomedicine and biotechnology. The ecological adaptability and high reproductive capacity of *H. illucens* make it a scalable and low-cost biofactory for AMP production. This aligns with global sustainability goals, promoting insect biotechnology as a viable route for future antimicrobial development. By characterizing the inducible peptide repertoire and its predicted functionalities, this study contributes to the identification of candidates suitable for translational application—including next-generation antimicrobial agents, biopreservatives, and immune-enhancing formulations, potentially applicable across clinical, veterinary, and agro-industrial contexts.

Future directions should include chemical synthesis and structural characterization of selected peptides, mechanistic studies of membrane interaction or intracellular targets, toxicity profiling in mammalian systems, and efficacy validation in infection models.

Additionally, transcriptomic profiling under varied infection conditions may help elucidate the upstream regulatory pathways governing AMP expression, offering new targets for biotechnological enhancement of peptide yield and specificity.

In conclusion, *H. illucens* emerges as a promising, sustainable, and versatile biotechnological platform for the discovery and scalable production of antimicrobial compounds. Its inducible immunopeptidome, analyzed through an integrated experimental and computational lens, represents a valuable resource for addressing the urgent need for novel therapeutics in the post-antibiotic era.

## Figures and Tables

**Table 1:** Concentrations of the samples obtained via precipitation with organic solvents from hemolymph.

Uninfected Larvae	Larvae Infected with *E. coli*	Larvae Infected with *M. flavus*
1.05 μg/μL	1.57 μg/μL	1.84 μg/μL

## Data Availability

The data that supports the findings of this study are available in the Supplementary Material of this article. Proteomics raw data are available via ProteomeXchange Consortium with identifier PXD071144.

## Supplementary Materials

The following supporting information can be downloaded at: https://www.mdpi.com/article/10.3390/insects17050486/s1, Supplementary Figures S1–S8: Antibacterial activity, assessed by agar diffusion assay, of HPLC fractions obtained from peptide extracts of uninfected larvae (A), larvae infected with *Escherichia coli* (B), and larvae infected with *Micrococcus flavus* (C), tested against different bacterial strains, including *E. coli*, *M. flavus*, *Pseudomonas aeruginosa*, *Staphylococcus aureus*, *Enterococcus faecalis*, *Salmonella* Typhimurium, enteroinvasive *E. coli* (EIEC), and carbapenem-resistant *Klebsiella pneumoniae* (CRKP); Supplementary Table S1: Table of protein identified in the SDS-PAGE analysis. For each protein identified the accession, description, frame and molecular weight were reported associated with the peptides identified. For each protein, the HPLC fraction of identification and the protein sequence are also reported; Supplementary Table S2: Proteins identified in the Differential Proteomics analysis. For each protein accession, description, total and unique peptides, coverage were reported associated with the ANOVA q-value and the post hoc (Tukey’s) significant pairs and factors for each condition; Supplementary Table S3: Proteins identified by SDS-PAGE analysis of HPLC fractions from *Hermetia illucens* peptide extracts, including BLASTp and UniProt annotation, predicted signal and pro-peptide cleavage sites, physicochemical properties, and in silico antimicrobial activity predictions; Supplementary Table S4: Proteins identified in the Differential Proteomics analysis, including BLASTp and UniProt annotation, predicted signal and pro-peptide cleavage sites, physicochemical properties, and in silico antimicrobial activity predictions.

## References

[1] Naghavi M., Vollset S.E., Ikuta K.S., Swetschinski L.R., Gray A.P., Wool E.E., Aguilar G.R., Mestrovic T., Smith G., Han C. (2024). Global burden of bacterial antimicrobial resistance 1990–2021: A systematic analysis with forecasts to 2050. Lancet.

[2] World Health Organization (2024). WHO Bacterial Priority Pathogens List.

[3] Drayton M., Deisinger J.P., Ludwig K.C., Raheem N., Müller A., Schneider T., Straus S.K. (2021). Host defense peptides: Dual antimicrobial and immunomodulatory action. Int. J. Mol. Sci..

[4] Raheem N., Straus S.K. (2019). Mechanisms of action for antimicrobial peptides with antibacterial and antibiofilm functions. Front. Microbiol..

[5] Eleftherianos I., Zhang W., Heryanto C., Mohamed A., Contreras G., Tettamanti G., Wink M., Bassal T. (2021). Diversity of insect antimicrobial peptides proteins—A functional perspective: A review. Int. J. Biol. Macromol..

[6] Huan Y., Kong Q., Mou H., Yi H. (2020). Antimicrobial peptides: Classification, design, application and research progress in multiple fields. Front. Microbiol..

[7] Manniello M.D., Moretta A., Salvia R., Scieuzo C., Lucchetti D., Vogel H., Sgambato A., Falabella P. (2021). Insect antimicrobial peptides: Potential weapons to counteract the antibiotic resistance. Cell. Mol. Life Sci..

[8] Moretta A., Scieuzo C., Salvia R., Popović Ž.D., Sgambato A., Falabella P. (2022). Tools in the era of multidrug resistance in bacteria: Applications for new antimicrobial peptides discovery. Curr. Pharm. Des..

[9] Moretta A., Moretta A., Salvia R., Scieuzo C., Di Somma A., Vogel H., Pucci P., Sgambato A., Wolff M., Falabella P. (2020). A bioinformatic study of antimicrobial peptides identified in the black soldier fly (*Hermetia illucens*) (Diptera: Stratiomyidae). Sci. Rep..

[10] Peng J., Li L., Wan Y., Yang Y., An X., Yuan K., Liang G. (2024). Molecular characterization and antimicrobial activity of cecropin family in *Hermetia illucens*. Dev. Comp. Immunol..

[11] Scieuzo C., Giglio F., Rinaldi R., Lekka M.E., Cozzolino F., Monaco V., Monti M., Salvia R., Falabella P. (2023). In vitro evaluation of the antibacterial activity of the peptide fractions extracted from the hemolymph of *Hermetia illucens* (Diptera: Stratiomyidae). Insects.

[12] Di Somma A., Moretta A., Cane C., Scieuzo C., Salvia R., Falabella P., Duilio A. (2022). Structural and functional characterization of a novel recombinant antimicrobial peptide from *Hermetia illucens*. Curr. Issues Mol. Biol..

[13] Li Z., Mao R., Teng D., Hao Y., Chen H., Wang X., Wang X., Yang N., Wang J. (2017). Antibacterial and immunomodulatory activities of insect defensins-DLP2 and DLP4 against multidrug-resistant *Staphylococcus aureus*. Sci. Rep..

[14] Van Moll L., De Smet J., Paas A., Tegtmeier D., Vilcinskas A., Cos P., Van Campenhout L. (2022). In vitro evaluation of antimicrobial peptides from the black soldier fly (*Hermetia illucens*) against a selection of human pathogens. Microbiol. Spectr..

[15] Lucchetti D., Rinaldi R., Artemi G., Salvia R., De Stefano F., Scieuzo C., Falabella P., Sgambato A. (2025). Peptide fractions extracted from the hemolymph of *Hermetia illucens* inhibit growth and motility and enhance the effects of traditional chemotherapeutics in human colorectal cancer cells. Int. J. Mol. Sci..

[16] Moretta A., Scieuzo C., Petrone A.M., Salvia R., Manniello M.D., Franco A., Lucchetti D., Vassallo A., Vogel H., Sgambato A. (2021). Antimicrobial peptides: A new hope in biomedical and pharmaceutical fields. Front. Cell. Infect. Microbiol..

[17] Rajasekhar N., Ramesh N., Prashantha C.N. (2020). Isolation and characterization of *Hermetia illucens* larval protein for the assessment of inhibitory activity against MCF7 and HeLa cell lines. Int. J. Innov. Technol. Explor. Eng..

[18] Rinaldi R., Laurino S., Salvia R., Russi S., De Stefano F., Galasso R., Sgambato A., Scieuzo C., Falco G., Falabella P. (2025). Biological activity of peptide fraction derived from *Hermetia illucens* L. (Diptera: Stratiomyidae) larvae haemolymph on gastric cancer cells. Int. J. Mol. Sci..

[19] Scieuzo C., Salvia R., Franco A., Pezzi M., Cozzolino F., Chicca M., Scapoli C., Vogel H., Monti M., Ferracini C. (2021). An integrated transcriptomic and proteomic approach to identify the main *Torymus sinensis* venom components. Sci. Rep..

[20] Hogsette J.A. (1992). New diets for production of house flies and stable flies (Diptera: Muscidae) in the laboratory. J. Econ. Entomol..

[21] Scieuzo C., Franco A., Salvia R., Triunfo M., Addeo N.F., Vozzo S., Piccolo G., Bovera F., Ritieni A., Di Francia A. (2023). Enhancement of fruit byproducts through bioconversion by *Hermetia illucens* (Diptera: Stratiomyidae). Insect Sci..

[22] Dang X.L., Tian J.H., Yi H.Y., Wang W.X., Zheng M., Li Y.F., Cao Y., Wen S.Y. (2006). Inducing and isolation of antibacterial peptides from oriental fruit fly, *Bactrocera dorsalis* Hendel. Insect Sci..

[23] Elhag O., Zhou D., Song Q., Soomro A.A., Cai M., Zheng L., Yu Z., Zhang J. (2017). Screening, expression, purification and functional characterization of novel antimicrobial peptide genes from *Hermetia illucens* (L.). PLoS ONE.

[24] Bradford M.M. (1976). A rapid and sensitive method for the quantitation of microgram quantities of protein utilizing the principle of protein-dye binding. Anal. Biochem..

[25] Hossain T.J. (2024). Methods for screening and evaluation of antimicrobial activity: A review of protocols, advantages, and limitations. Eur. J. Microbiol. Immunol..

[26] Clinical and Laboratory Standards Institute (CLSI) (2024). Performance Standards for Antimicrobial Disk Susceptibility Tests.

[27] Balouiri M., Sadiki M., Ibnsouda S.K. (2016). Methods for in vitro evaluating antimicrobial activity: A review. J. Pharm. Anal..

[28] Gallagher S.R. (2012). SDS-polyacrylamide gel electrophoresis (SDS-PAGE). Curr. Protoc. Essent. Lab. Tech..

[29] Iaconis D., Monti M., Renda M., van Koppen A., Tammaro R., Chiaravalli M., Cozzolino F., Pignata P., Crina C., Pucci P. (2017). The centrosomal OFD1 protein interacts with the translation machinery and regulates the synthesis of specific targets. Sci. Rep..

[30] Andolfo I., Monaco V., Cozzolino F., Rosato B.E., Marra R., Cerbone V., Pinto V.M., Forni G.L., Unal S., Iolascon A. (2023). Proteome alterations in erythrocytes with PIEZO1 gain-of-function mutations. Blood Adv..

[31] Cozzolino F., Canè L., Gatto M.C., Iacobucci I., Sacchettino L., De Biase D., Di Napoli E., Paciello O., Avallone L., Monti M. (2023). Proteomic signature profiling in the cortex of dairy cattle unravels the physiology of brain aging. Front. Aging Neurosci..

[32] Cozzolino F., Canè L., Sacchettino L., Gatto M.C., Iacobucci I., Gatta C., De Biase D., Di Napoli E., Paciello O., Avallone L. (2023). Preliminary evaluation of the proteomic profiling in the hippocampus of aged grazing cattle. Front. Aging Neurosci..

[33] Duckert P., Brunak S., Blom N. (2004). Prediction of proprotein convertase cleavage sites. Protein Eng. Des. Sel..

[34] Wang Z., Wang G. (2004). APD: The antimicrobial peptide database. Nucleic Acids Res..

[35] Wang G., Li X., Wang Z. (2009). APD2: The updated antimicrobial peptide database and its application in peptide design. Nucleic Acids Res..

[36] Wang G., Li X., Wang Z. (2016). APD3: The antimicrobial peptide database as a tool for research and education. Nucleic Acids Res..

[37] Bjellqvist B., Hughes G.J., Pasquali C., Paquet N., Ravier F., Sanchez J.C., Frutiger S., Hochstrasser D.F. (1993). The focusing positions of polypeptides in immobilized pH gradients can be predicted from their amino acid sequences. Electrophoresis.

[38] Bjellqvist B., Basse B., Olsen E., Celis J.E. (1994). Reference points for comparisons of two-dimensional maps of proteins from different human cell types defined in a pH scale where isoelectric points correlate with polypeptide compositions. Electrophoresis.

[39] Gasteiger E., Hoogland C., Gattiker A., Duvaud S., Wilkins M.R., Appel R.D., Bairoch A., Walker J.M. (2005). Protein identification and analysis tools on the ExPASy server. The Proteomics Protocols Handbook.

[40] Gawde U., Chakraborty S., Waghu F.H., Barai R.S., Khanderkar A., Indraguru R., Shirsat T., Idicula-Thomas S. (2023). CAMPR4: A database of natural and synthetic antimicrobial peptides. Nucleic Acids Res..

[41] Sievers F., Wilm A., Dineen D., Gibson T.J., Karplus K., Li W., Lopez R., McWilliam H., Remmert M., Söding J. (2011). Fast, scalable generation of high-quality protein multiple sequence alignments using Clustal Omega. Mol. Syst. Biol..

[42] Bertini L., Cozzolino F., Proietti S., Falconieri G.S., Iacobucci I., Salvia R., Caruso C. (2021). What Antarctic plants can tell us about climate changes: Temperature as a driver for metabolic reprogramming. Biomolecules.

[43] Zhang J., Li J., Peng Y., Gao X., Song Q., Zhang H., Zhang J. (2022). Structural and functional characterizations and heterogenous expression of the antimicrobial peptides, Hidefensins, from black soldier fly, *Hermetia illucens* (L.). Protein Expr. Purif..

[44] Park S.I., Kim J.W., Yoe S.M. (2015). Purification and characterization of a novel antibacterial peptide from black soldier fly (*Hermetia illucens*) larvae. Dev. Comp. Immunol..

[45] Li B., Yang N., Wang X., Hao Y., Mao R., Li Z., Wang Z., Teng D., Wang J. (2020). An enhanced variant designed from DLP4 cationic peptide against *Staphylococcus aureus* CVCC 546. Front. Microbiol..

[46] Jiang Y., Li X., Lin Y. (2022). Production of antimicrobial peptide DLP4 in *Escherichia coli* using an ELP-intein system. Chin. J. Biotechol..

[47] Derin E., Van Moll L., Wouters M., De Vooght L., De Stefano F., Scieuzo C., Falabella P., Cos P. (2026). Antimicrobial effects of novel *Hermetia illucens* peptides. Sci. Rep..

