# Supplementary material for: A Multidisciplinary Integrated Approach for the Identification and Characterization of the AMP Profile in Hermetia illucens Hemolymph
Source: Insects. 2026 May 9;17(5):486. doi: 10.3390/insects17050486 (PMC13207985; doi:10.3390/insects17050486)
Supplement: Supplementary file 1 [file insects-17-00486-s001.zip › De Stefano et al., 2026_Supplementary Figures.pdf]

Supplementary Figures S1-S8: Antibacterial activity, assessed by agar diffusion assay, of HPLC fractions obtained from peptide extracts of uninfected larvae (A), larvae infected with *Escherichia coli* (B), and larvae infected with *Micrococcus flavus* (C), tested against different bacterial strains, including *E. coli*, *M. flavus*, *Pseudomonas aeruginosa*, *Staphylococcus aureus*, *Enterococcus faecalis*, *Salmonella* Typhimurium, enteroinvasive *E. coli* (EIEC), and carbapenem-resistant *Klebsiella pneumoniae* (CRKP).

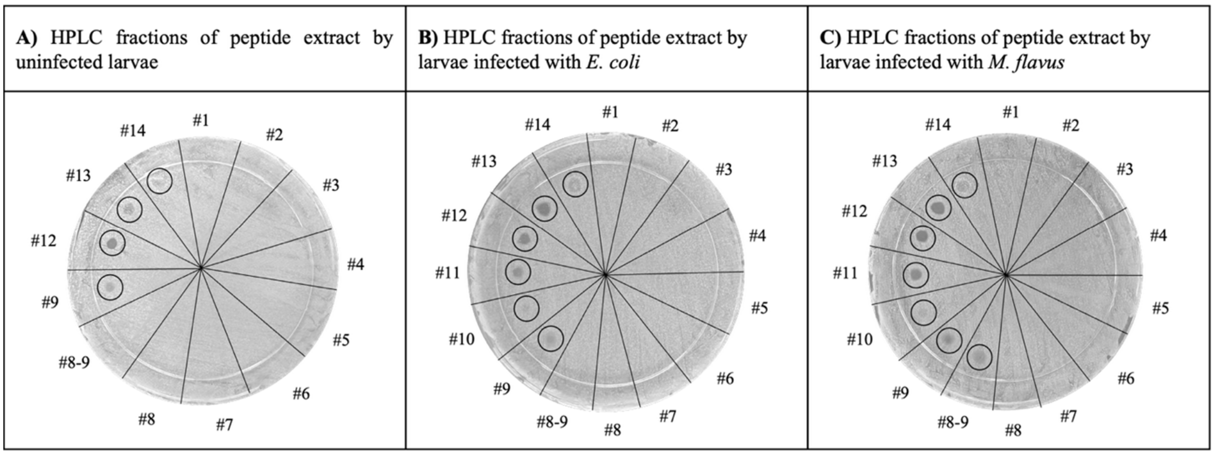

Supplementary Figure S1. Agar diffusion assay against *E. coli* of HPLC fractions of peptide extracts by uninfected larvae (A), HPLC fractions of peptide extract by larvae infected with *E. coli* (B), and HPLC fractions of peptide extract by larvae infected with *M. flavus* (C). Each spot was scored qualitatively as “inhibition” or “no inhibition” according to the criteria detailed in Section 2.7.

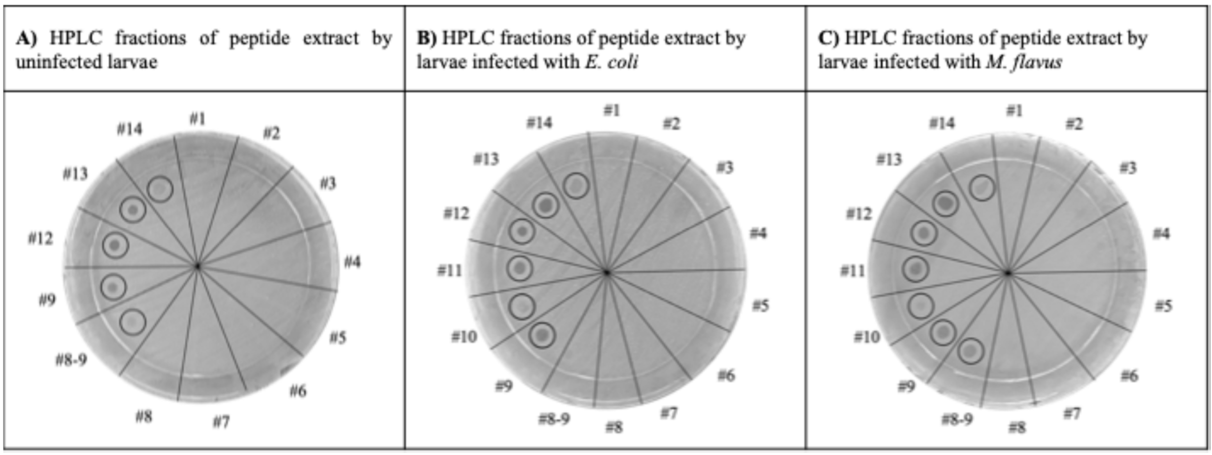

Supplementary Figure S2. Agar diffusion assay against *M. flavus* of HPLC fractions of peptide extracts by uninfected larvae (A), HPLC fractions of peptide extract by larvae infected with *E. coli* (B), and HPLC fractions of peptide extract by larvae infected with *M. flavus* (C). Each spot was scored qualitatively as “inhibition” or “no inhibition” according to the criteria detailed in Section 2.7.

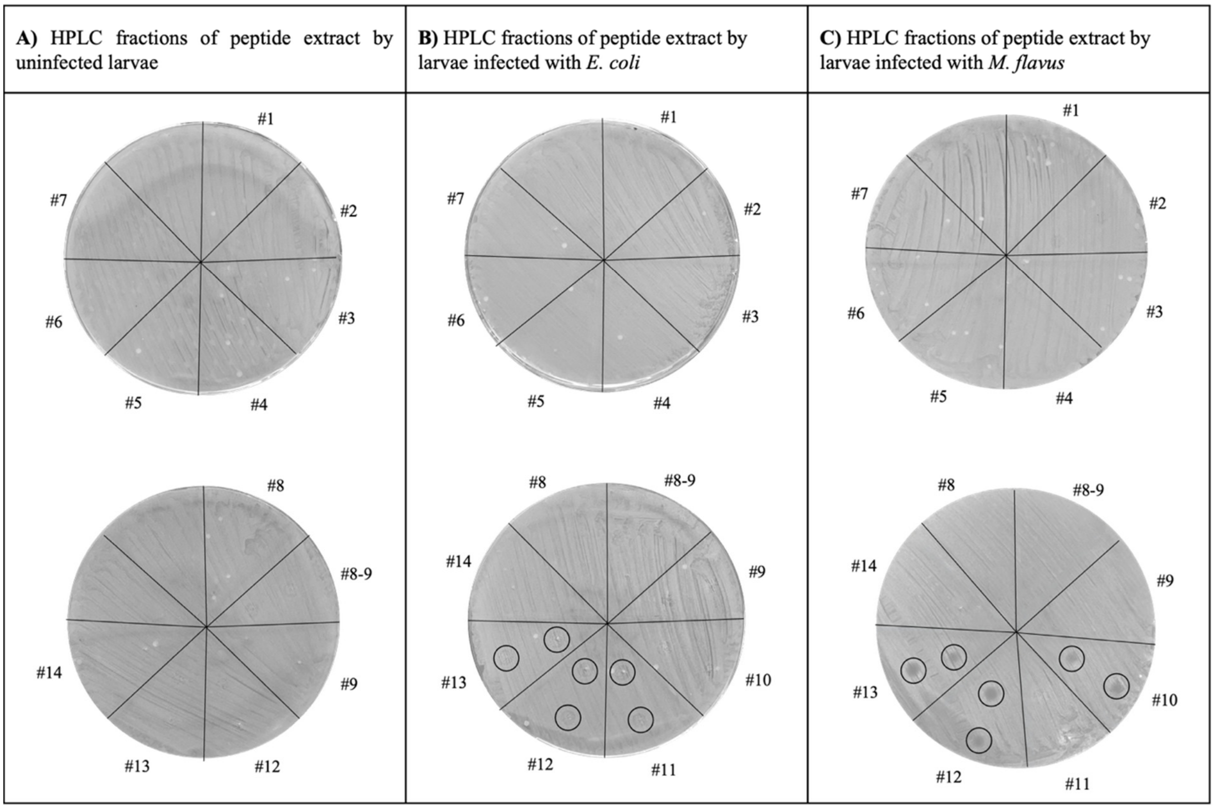

Supplementary Figure S3. Agar diffusion assay against *P. aeruginosa* of HPLC fractions of peptide extracts by uninfected larvae (A), HPLC fractions of peptide extract by larvae infected with *E. coli* (B), and HPLC fractions of peptide extracts by larvae infected with *M. flavus* (C). Each spot was scored qualitatively as “inhibition” or “no inhibition” according to the criteria detailed in Section 2.7.

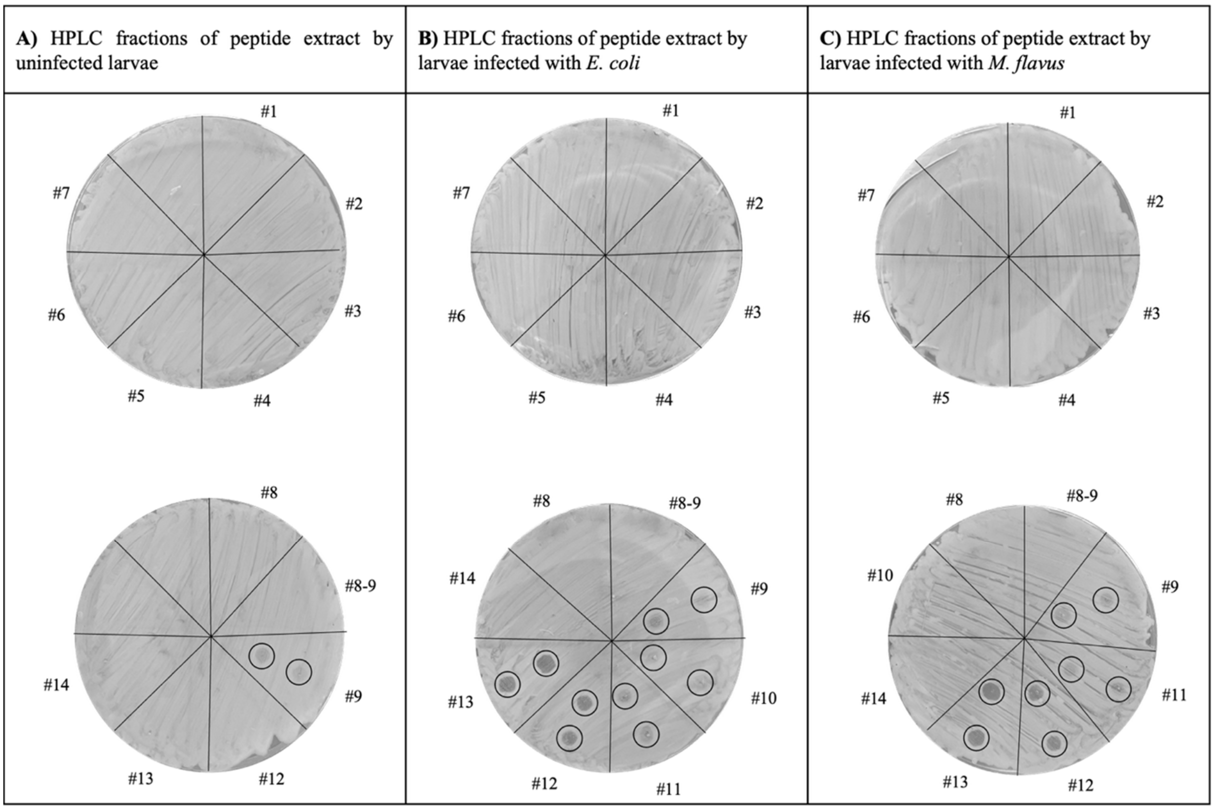

Supplementary Figure S4. Agar diffusion assay against *S. aureus* of HPLC fractions of peptide extracts by uninfected larvae (A), HPLC fractions of peptide extract by larvae infected with *E. coli* (B), and HPLC fractions of peptide extract

by larvae infected with *M. flavus* (C). Each spot was scored qualitatively as “inhibition” or “no inhibition” according to the criteria detailed in Section 2.7.

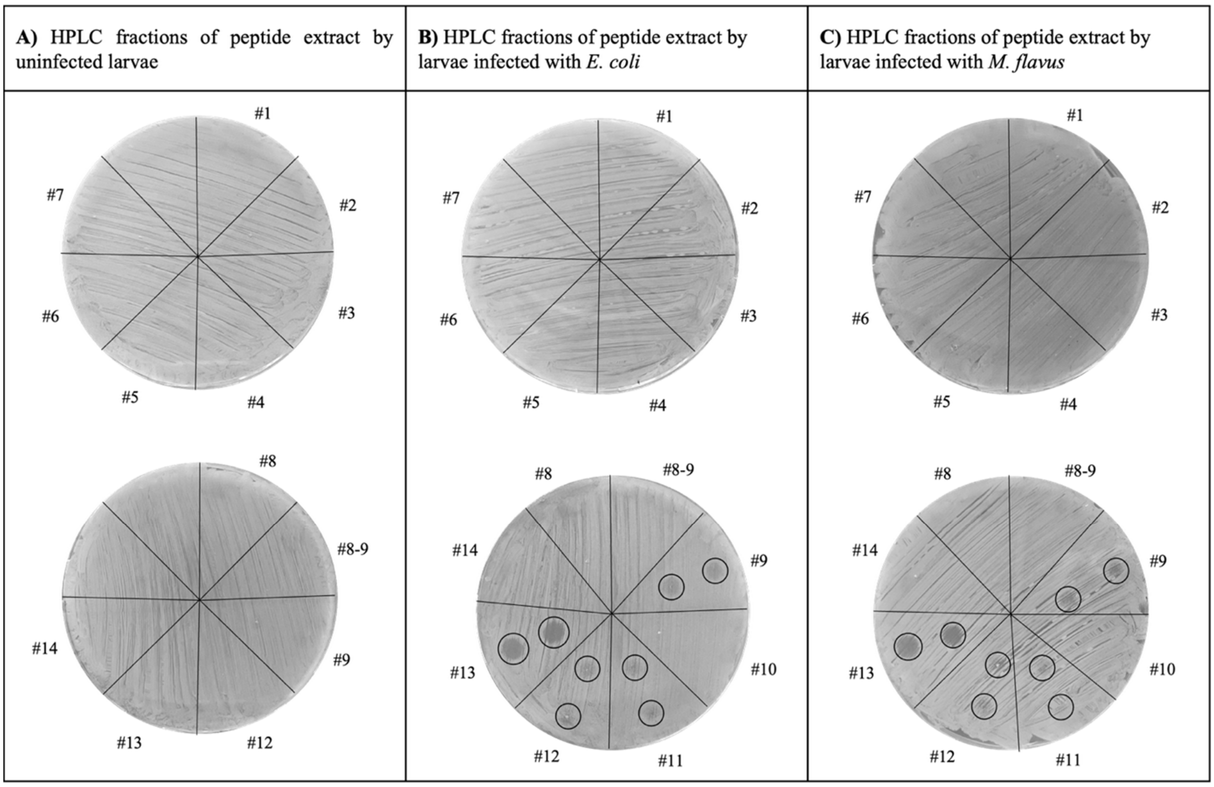

Supplementary Figure S5. Agar diffusion assay against *E. faecalis* of HPLC fractions of peptide extracts by uninfected larvae (A), HPLC fractions of peptide extract by larvae infected with *E. coli* (B), and HPLC fractions of peptide extract by larvae infected with *M. flavus* (C). Each spot was scored qualitatively as “inhibition” or “no inhibition” according to the criteria detailed in Section 2.7.

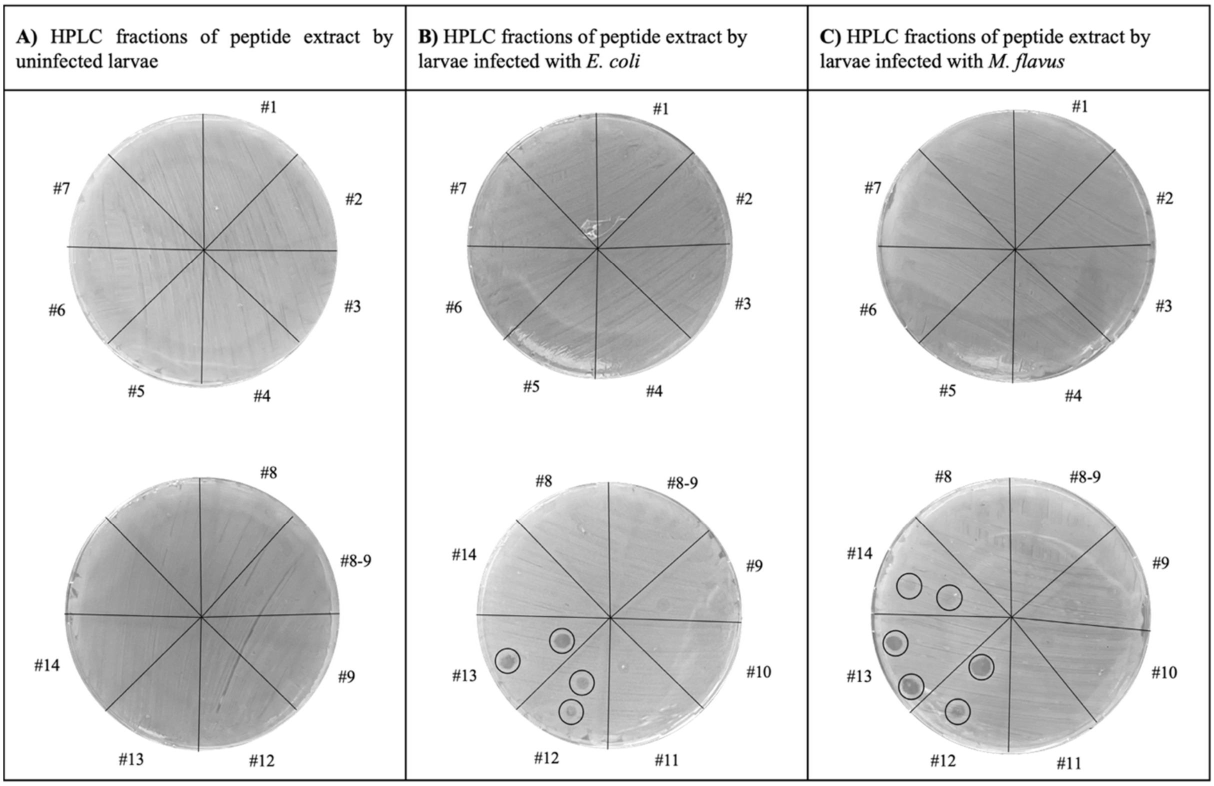

Supplementary Figure S6. Agar diffusion assay against *S.* Typhimurium of HPLC fractions of peptide extracts by uninfected larvae (A), HPLC fractions of peptide extract by larvae infected with *E. coli* (B), and HPLC fractions of peptide extract by larvae infected with *M. flavus* (C). Each spot was scored qualitatively as "inhibition" or "no inhibition" according to the criteria detailed in Section 2.7.

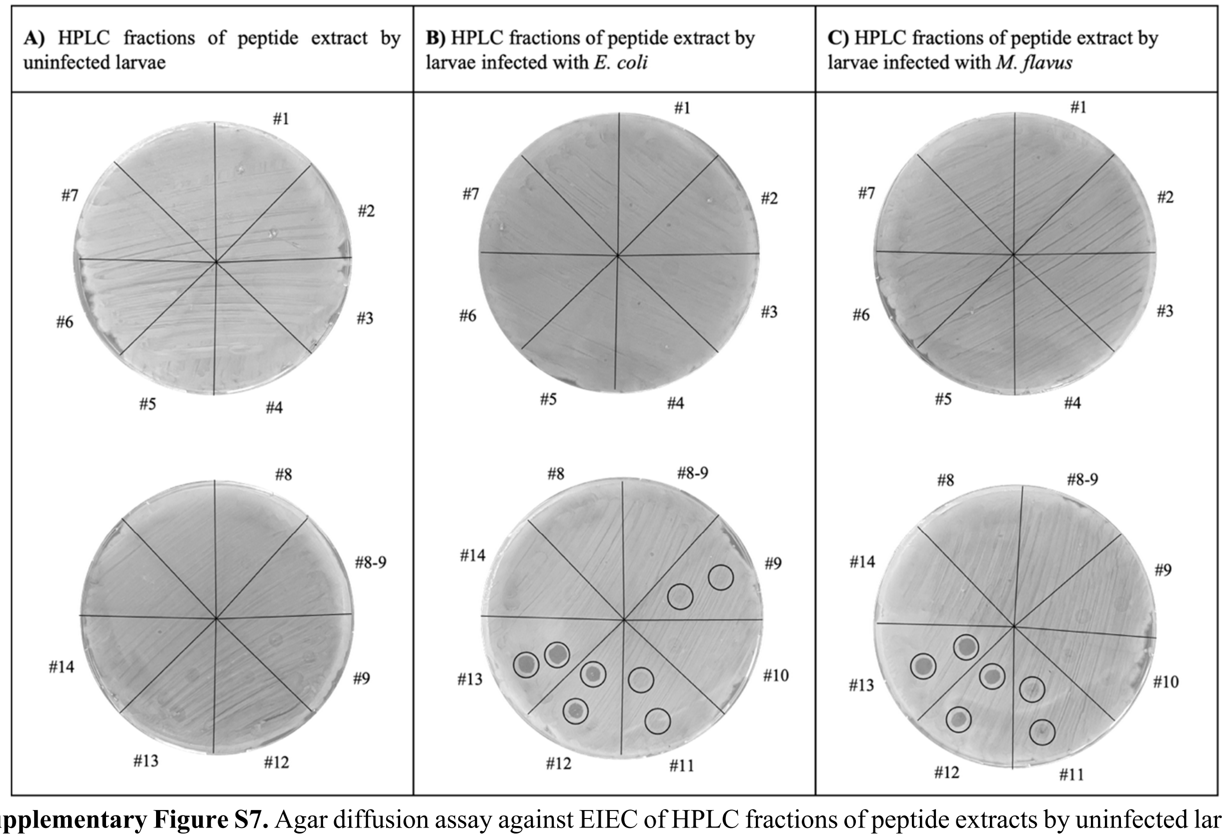

Supplementary Figure S7. Agar diffusion assay against EIEC of HPLC fractions of peptide extracts by uninfected larvae (A), HPLC fractions of peptide extract by larvae infected with *E. coli* (B), and HPLC fractions of peptide extract by larvae

infected with *M. flavus* (C). Each spot was scored qualitatively as “inhibition” or “no inhibition” according to the criteria detailed in Section 2.7.

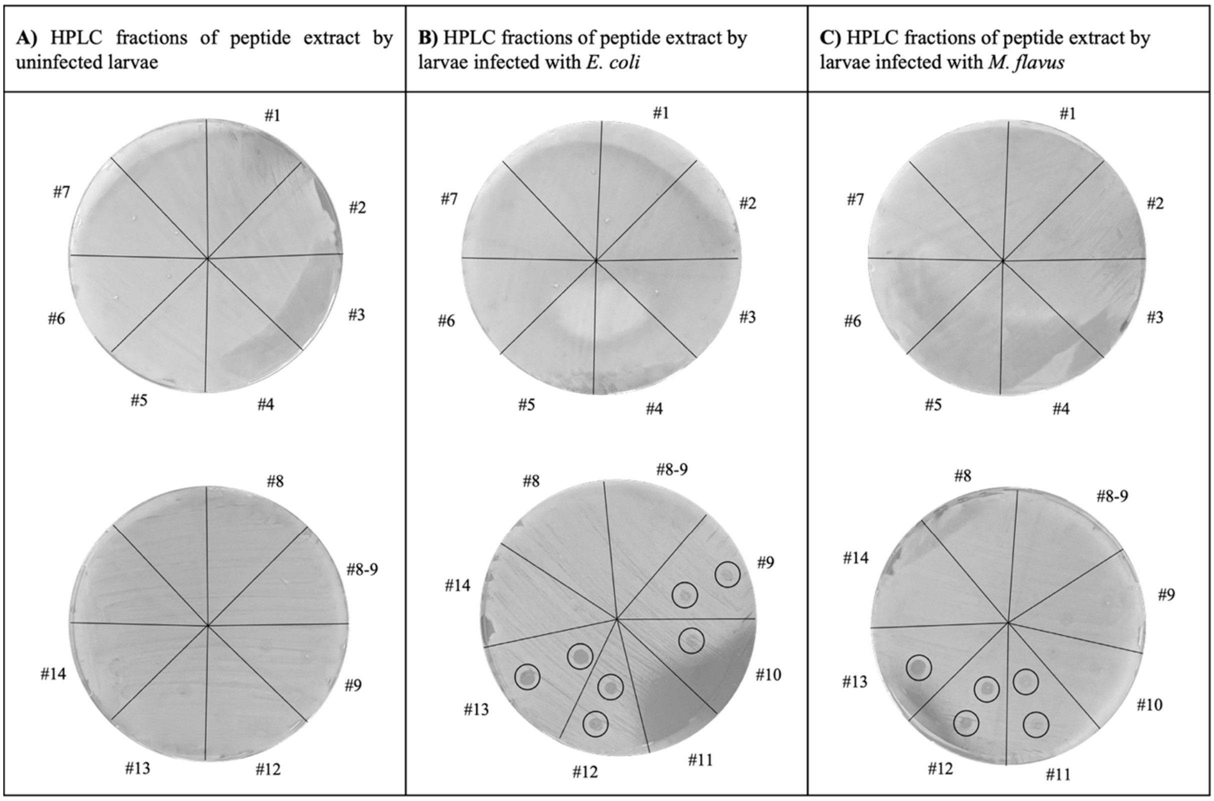

Supplementary Figure S8. Agar diffusion assay against CRKP of HPLC fractions of peptide extracts by uninfected larvae (A), HPLC fractions of peptide extract by larvae infected with *E. coli* (B), and HPLC fractions of peptide extract by larvae infected with *M. flavus* (C). Each spot was scored qualitatively as “inhibition” or “no inhibition” according to the criteria detailed in Section 2.7.
